# A Combination of Nutriments Improves Mitochondrial Biogenesis and Function in Skeletal Muscle of Type 2 Diabetic Goto–Kakizaki Rats

**DOI:** 10.1371/journal.pone.0002328

**Published:** 2008-06-04

**Authors:** Weili Shen, Jiejie Hao, Chuan Tian, Jinmin Ren, Lu Yang, Xuesen Li, Cheng Luo, Carl W. Cotma, Jiankang Liu

**Affiliations:** 1 Institute for Nutritional Science, Shanghai Institutes of Biological Sciences, Chinese Academy of Sciences, Shanghai, China; 2 Graduate School of the Chinese Academy of Sciences, Beijing, China; 3 Institute for Brain Aging and Dementia, University of California Irvine, Irvine, California, United States of America; Canadian Agency for Drugs and Technologies in Health, Canada

## Abstract

**Background:**

Recent evidence indicates that insulin resistance in skeletal muscle may be related to reduce mitochondrial number and oxidation capacity. However, it is not known whether increasing mitochondrial number and function improves insulin resistance. In the present study, we investigated the effects of a combination of nutrients on insulin resistance and mitochondrial biogenesis/function in skeletal muscle of type 2 diabetic Goto–Kakizaki rats.

**Methodology/Principal Findings:**

We demonstrated that defect of glucose and lipid metabolism is associated with low mitochondrial content and reduced mitochondrial enzyme activity in skeletal muscle of the diabetic Goto-Kakizaki rats. The treatment of combination of R-α-lipoic acid, acetyl-L-carnitine, nicotinamide, and biotin effectively improved glucose tolerance, decreased the basal insulin secretion and the level of circulating free fatty acid (FFA), and prevented the reduction of mitochondrial biogenesis in skeletal muscle. The nutrients treatment also significantly increased mRNA levels of genes involved in lipid metabolism, including peroxisome proliferator–activated receptor-α (*Pparα*), peroxisome proliferator–activated receptor-*δ (Pparδ)*, and carnitine palmitoyl transferase-1 *(Mcpt-1)* and activity of mitochondrial complex I and II in skeletal muscle. All of these effects of mitochondrial nutrients are comparable to that of the antidiabetic drug, pioglitazone. In addition, the treatment with nutrients, unlike pioglitazone, did not cause body weight gain.

**Conclusions/Significance:**

These data suggest that a combination of mitochondrial targeting nutrients may improve skeletal mitochondrial dysfunction and exert hypoglycemic effects, without causing weight gain.

## Introduction

Increasing evidence suggests that mitochondrial dysfunction due to oxidative damage is a major contributor to aging, degenerative diseases such as cancer, and metabolic syndrome, such as obesity and type 2 diabetes [1], [2], [3]. Skeletal muscle insulin resistance may play an important role in the pathogenesis of the metabolic syndrome and 2 type diabetes [4]. Recent studies reported insulin resistance is associated with impaired skeletal muscle oxidation capacity and reduced mitochondrial number and function [5], [6]. Therefore, protecting mitochondria from oxidative damage to improve mitochondrial function in skeletal muscle seems a possible strategy to prevent and treat diseases associated with mitochondrial dysfunction [7], [8], [9], 10. For example, rosiglitazone improves the suppression of adipose mitochondrial biogenesis in db/db and high fat diet-fed mice [11]. Pioglitazone reduces hyperglycemia, hyperlipidemia, and hyperinsulinemia in male fatty rats [12], improves mitochondrial function and stimulates mitochondrial biogenesis in human adipocyte/tissue in vitro [13], [14] or human neuron-like cells [15]. Metformin delays the manifestation of diabetes and vascular dysfunction and reduces mitochondrial oxidative stress in Goto-Kakizaki (GK) rats [16]. However, the use of any pharmacological therapy for type 2 diabetes, such as thiazolidinedione, insulin, metformin, and other oral hypoglycemic agents or combination therapy with or without insulin appears to be associated with an increased risk of heart failure and body weight gain [17]. Therefore, effective treatments without apparent side effects are greatly needed for preventing and treating diabetes and other metabolic syndromes.

We have defined a group of mitochondrial targeting antioxidants/metabolites as mitochondrial nutrients [8], [9], [18], i.e., nutrients which improve mitochondrial function and protect mitochondria from oxidative damage, including those that can 1) inhibit or prevent oxidant production in mitochondria; 2) scavenge and inactivate free radicals and reactive oxygen species; 3) repair mitochondrial damage and enhance antioxidant defenses by stimulating mitochondrial biogenesis and inducing phase-2 enzymes; and 4) act as cofactors/substrates to protect mitochondrial enzymes and/or stimulate enzyme activity. One good example of mitochondrial nutrients is R-α-lipoic acid (LA) [9], [19], [20], [21].

More recently, we have examined the effect of LA and acetyl-L-carnitine (ALC), as well as their combination, on mitochondrial biogenesis in 3T3-L1 adipocyte. We found that treatment with a combination of LA and ALC significantly improved mitochondrial function and increased mitochondrial biogenesis related transcription factors while the treatments with only LA and ALC alone at the same concentrations showed little effect [22]. From these results, we have concluded that the combination of mitochondrial targeting nutrients may complementarily promote mitochondrial synthesis and adipocyte metabolism and possibly, like thiazolidinedione drugs, prevent and treat insulin resistance in type 2 diabetes. In the present study, we investigated the effects of a combination of LA and ALC, with two other mitochondrial nutrients, nicotinamide and biotin, on improving glucose tolerance, insulin release, fatty acid metabolism, and mitochondrial biogenesis and function in the spontaneous diabetic GK rats.

## Results

### Metabolic characteristics of GK rats

The metabolic characteristics of GK rats are summarized in Table 1. After the 12-week administration, there was no difference in body weight between the untreated and the nutrients-treated GK rats, however, the body weight in the pioglitazone-treated group was significantly higher than that of other groups. The pioglitazone-induced gain of body weight is consistent with a previous report [23]. The pioglitazone treatment tended to increase food intake (measured for individual rats in g/kg/24 h) and significantly reduced the levels of triglyceride and total cholesterol in blood. The nutrients-treatment did not affect fasting glucose and triglyceride levels, but significantly reduced fasting plasma insulin (p<0.01 vs. GK) and slightly decreased total cholesterol level in GK rats.

**Table 1:** Morphometric and plasma variables in GK rats with different treatments.

	GK	GK with nutrients	GK with pioglitazone
Body weight (g)	326.5±26.0	329.2±19.8	419.3±21.6 **
Food intake (g/kg/d)	81.5±6.5	78.1.4±11.1	84.8±12.4
Fasting plasma glucose (mmol/l)	6.0±0.15	5.6±0.16	5.8±0.23
Fasting plasma insulin (ng/ml)	1.06±0.14	0.60±0.08*	0.99±0.07
Triglycerides (mmol/l)	1.83±0.04	1.86±0.23	0.83±0.04**
Total cholesterol (mmol/l)	3.06±0.06	2.87±0.15	2.59±0.04 *

Values are mean±SEM of 12 animals in each group.

* p<0.05 vs. GK control;

** p<0.01 vs. GK control.

### Effects on glucose tolerance and plasma FFA

**Figure 1:**
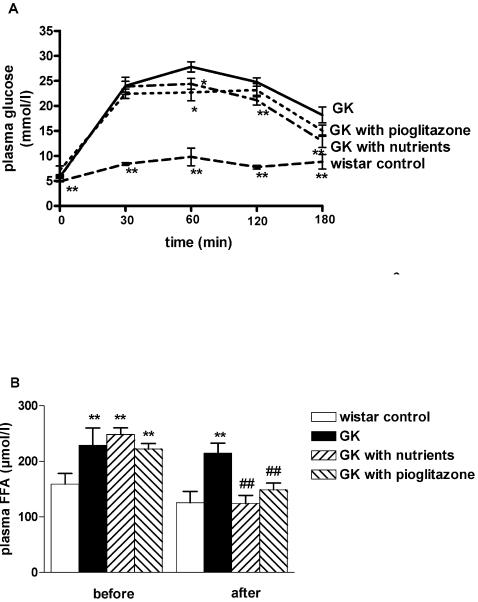
Effects of mitochondrial nutriments on OGTT and plasma free fatty acid (FFA). A: OGTT was carried out at the end of 12 weeks of nutrient administration. All rats fasted overnight before OGTT. Blood was taken from the retrobulbar vein at 0, 30, 60, 120 and 180 min after the oral glucose administration (5 g/kg body weight). Plasma glucose concentration was determined by the glucose oxidase method. Data are means±S.E. of 12 observations in each group. * p<0.05, **p<0.01 vs. the respective values in the GK control group. B: Plasma free fatty acid was measured in groups at beginning or after nutrient administration for 12 weeks. Serum levels of free fatty acids in different groups after overnight fasting. Data are means±S.E. of 12 animals in each group. **p<0.01 vs. the respective values in the Wistar control group. ##p<0.01 vs. the respective values in the GK control group.

We first examined whether the mitochondrial nutrient treatments could improve the glucose tolerance because hyperglycemia is the defining characteristic of type 2 diabetes and improvement of glucose tolerance is one of the most critical criteria for evaluating the effectiveness of hypoglycemic drugs. Fig 1A illustrates changes in the blood glucose levels during OGTT in GK and Wistar rats. Hyperglycemic responses in GK rats to OGTT were significantly greater than those in Wistar rats. In the diabetic GK rats treated with nutriments for 12 week, the levels of glucose obtained 60, 120 and 180 min after glucose intake were significantly lower than that in the untreated GK diabetic rats (24.4±1.0 mmol/l vs. 27.8±1.0 mmol/l, p<0.05; 21.1±0.9 mmol/l vs. 24.7±0.9 mmol/l, p<0.01 and 12.9±1.3 mmol/l vs. 15.0±1.5 mmol/l, p<0.01, respectively). The blood glucose level at 60 min in the pioglitazone-treated diabetic rats was also significantly lower than the corresponding control diabetic value (22.6±1.6 mmol/l vs.27.8±1.0 mmol/l, p<0.05) (Figure 1A).

Chronic elevation in plasma FFA levels is commonly associated with impaired insulin-mediated glucose uptake in skeletal muscles and often coexists with obesity and type 2 diabetes. The higher FFA may cause peripheral insulin resistance by interfering with the access of insulin to skeletal muscle or the insulin signaling resulting in reduced glucose transport into muscle [24]. Therefore, lowering FFA has been postulated to be a potential therapeutic target for type 2 diabetes. In our results, we have found that the plasma FFA in the GK diabetic rats was significantly higher than those in the non-diabetic Wistar rats before treatments. After 12 week treatment, the plasma FAA was significantly decreased in all groups with drugs. The increases in plasma FFA in the untreated GK diabetic rats were still significantly higher than those in the non-diabetic Wistar rats (Fig 1B).

### Effects on mitochondrial DNA and protein in soleus muscle

**Figure 2:**
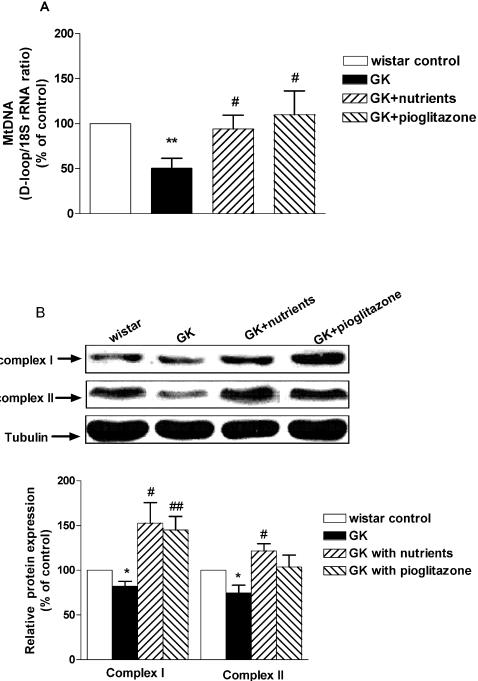
Effect of mitochondrial nutriments on mitochondrial DNA and protein in soleus muscle. A: Total DNA was isolated from soleus muscle. The mtDNA contents were determined by real-time PCR. The DNA contents of mtDNA and nuclear 18S rRNA gene (18S rDNA) were calculated from the standard curve and the relative ratios of mtDNA contents against nuclear 18S rRNA gene were determined in each group. Results are expressed as percentage of Wistar control. Data are mean+SEM (n = 5). **P<0.01 vs. Wistar control; # p<0.05 vs. GK control. B: Protein (10 µg) was solubilized in SDS sample buffer and analyzed by western blotting with antibodies against tubulin, mitochondrial electron transport complex I and complex II. The quantitative analyses of the bands by densitometry are shown. Results are presented as percentage of Wistar control. *p<0.05 vs. Wistar control; #P<0.05, ##p<0.01 vs. GK control.

D-loop is known as the major site of transcription initiation on both the heavy and light strands of mtDNA. In Fig 2A, the ratio of mtDNA D-loop/nuclear DNA 18S rRNA in GK rats was significantly lower than that in Wistar rats (p<0.01vs.wistar control). The pioglitazone-treated diabetic rats was significantly higher than the untreated GK diabetic rats (p<0.05 vs. control). In the diabetic rats treated with nutriments for 12 week, the ratio of mt D-loop/18S rRNA was significantly higher than the untreated GK diabetic rats (p<0.05 vs. GK control).

If there is an increased mitochondrial DNA content, there should be a concomitant increase in viable mitochondria and mitochondrial components, including electron chain transport complex enzymes. We examined the effects of mitochondrial nutrients on the expression of complex I and II and found that the expression of complex I and complex II (Fig 2B) in GK rats were significantly lower than Wistar rats (82±4.2% p<0.05 vs. Wistar control; 74.5±7.0% p<0.05 vs. Wistar control; respectively). In the diabetic GK rats treated with nutriments, the expression of complex I and complex II were increased significantly compared with that in the untreated GK rats (152±18%, p<0.05 vs. GK control; 121±6.7%, p<0.05 vs. GK control; respectively). The expression of complex I, but not complex II, in the pioglitazone-treated GK rats was also significantly higher than the GK control rats (144±12.6%. p<0.01vs.GK control).

### Effects on mitochondrial complex activities

**Figure 3:**
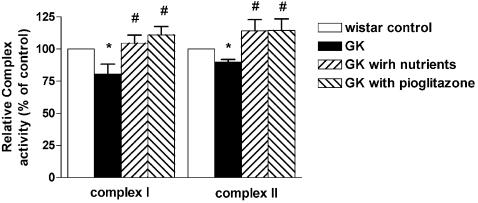
Effect of mitochondrial nutriments on complex I and II enzyme activity in soleus muscle. After 12 week treatment, mitochondria were isolated from soles and the activities of mitochondrial Complex I and Complex II were assayed spectrometrically using the conventional assays. Results are presented as percentage of Wistar control. Data are means±SEM of 12 animals in each group. *p<0.05, **p<0.01 vs. Wistar control; #p<0.05 vs. GK control.

The improvement of the expression of the mitochondrial complex enzymes suggests there should be a consequent improvement of the complex enzyme activity in the GK rats. Our measurement showed that the activity of mitochondrial complex I and II (Fig 3) were significantly lower in GK rats than in Wister rats (80.5±6.3%, p<0.05 vs. control; 89.9±1.9%, p<0.01 vs. control; respectively) and the administration of mitochondrial nutriments and pioglitazone to the GK rats significantly increased the activity of complex I (104±5.0%, p<0.05 vs. GK control; 110±5.4%, p<0.05 vs. GK control, respectively) and the activity of complex II (114.2±7.1%, p<0.05 vs. GK control; 114.6±7.4%, p<0.05 vs. GK control, respectively).

### Effects on expression of PPARGC1A, Nrf1 and Tfam

**Figure 4:**
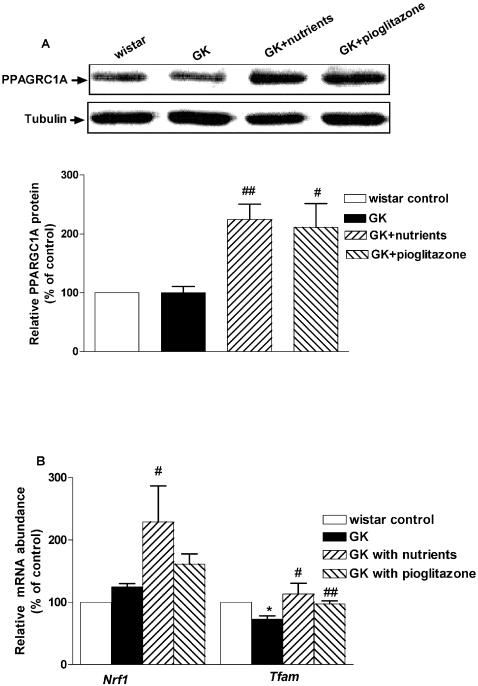
Effect of mitochondrial nutriments on expression of PPARGC1A, Nrf1 and Tfam. A: Western blot analysis in adipocytes of PPARGC1A. Quantitative values were tabulated with the ratio of densities of PPARGC1A: tubulin. Values are mean±SE of 12 animals in each group.*p<0.05, p<0.01 vs. Wistar control; # p<0.05, ##p<0.01 vs. GK control. B: Total RNA was isolated from soleus muscle. mRNA of *Nrf1* and *Tfam* were analyzed by means of quantitative RT-PCR with gene-specific oligonucleotide probes in muscle. The cycle number at which the various transcripts were detectable was compared to that of nuclear18S rRNA as an internal control, and expressed as arbitrary units versus values in Wistar control taken 100. All values are mean±SEM of 12 animals in each group.*p<0.05, ** p<0.01 vs. Wistar control; #p<0.05, ##p<0.01 vs. GK control.

Peroxisome proliferator activator protein-γ co-activator-1α (PPARGC1A) is a transcription coactivator that promotes mitochondrial biogenesis and mitochondrial fatty acid oxidation. Increasing evidence suggests that PPARGC1A is involved in the pathogenesis of type 2 diabetes by playing a pivotal role in the control of genetic pathways that result in homeostatic glucose utilization in liver and muscle, beta cell insulin secretion and mitochondrial biogenesis [25], [26]. Rosiglitazone was shown to restore PPARGC1A expression in obese patients with type 2 diabetes mellitus [27]. In our study, no difference in PPARGC1A expression was found between the Wistar and the untreated GK rats, however, both the treatment with pioglitazone (210±45%, p<0.01 vs. GK control) and mitochondrial nutriments (224±25%, p<0.01 vs. GK control) significantly increased the expression of PPARGC1A (Fig 4A).

Transcription factors nuclear respiratory factor-1 (*Nrf1)* and mitochondrial transcription factor A (*Tfam)* are involved in regulating expression of nuclear genes encoding major mitochondrial proteins that regulate mtDNA transcription and replication [9], [26]. We could not find difference in the expression of *Nrf1* mRNA between the Wistar and the untreated GK rats while the pioglitazone treatment resulted in a trend towards an increase and the mitochondrial nutriment administration significantly increased the mRNA expression of *Nrf1* (334±74.1, p<0.01vs.GK control) (Fig 4B). Levels of *Tfam* mRNA were found to be significantly lower in the GK rats than in the Wister rats (72.6±5.7%, p<0.01 vs. Wistar control). After the 12 week treatment, the expression of *Tfam* was significantly increased in the nutriments-treated group (113.3±17.5%, p<0.05 vs. GK control); and in the pioglitazone-treated group (97.4±5.0%, p<0.01 vs. GK control).

### Effects on expression of Pparα, Pparδ and Mcpt-1

**Figure 5:**
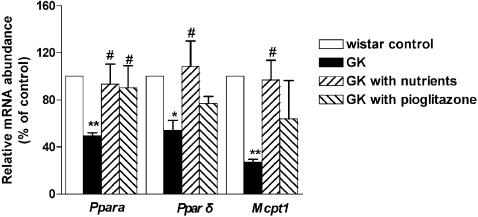
Effect of mitochondrial nutriments on expression of Pparα, Pparδ and Mcpt-1. Total RNA was isolated from soleus muscle. *Ppara, Pparδ* and *Mcpt-1* mRNA were analyzed by means of quantitative RT-PCR with gene-specific oligonucleotide probes in muscle. The cycle number at which the various transcripts were detectable was compared to that of nuclear 18S rRNA as an internal control, and expressed as arbitrary units versus values in Wistar control taken 100. All values are mean±SEM of 12 animals in each group. *p<0.05, ** p<0.01 vs. Wistar control; #p<0.05, ##p<0.01 vs. GK control.

*Pparα* and *Pparδ,* which are ligand-activated transcription factors, are critical to fat metabolism. *Pparα* mediates the hypotriglyceridemic effect of fibrates by inducing high rates of mitochondrial peroxisome β-oxidation in liver, kidney, heart and muscle, and by decreasing the plasma concentration of triacylglycerol-rich lipoproteins [28]. *Pparδ* is mainly expressed in brown adipose tissue and muscle, and specifically induces the expression of genes required for fatty acid oxidation and energy dissipation which lead to the improvement of lipid profiles and the reduction of adiposity [29]. Increasing the level of *Pparδ* in white adipose tissues has been suggested as a potential strategy to treat obesity [30]. As shown in Fig 5, levels of *Pparα* and *Pparδ* mRNA were significantly lower in GK rats than that in Wister rats (49.6±2.2%, p<0.01 vs. Wistar control; and 54.3±8.2%, p<0.05 vs. Wistar control, respectively). Administration of mitochondrial nutriments to the GK rats significantly increased the expression of *Pparα* (93±15.8%, p<0.05 vs. GK control) and *Pparδ* (108±21%, p<0.05 vs. GK control) in soleus muscle. The treatments with pioglitazone only significantly increased the expression of *Pparα* mRNA (90±19.7%, p<0.05 vs. GK control; respectively), but not *Pparδ* mRNA.

Muscle-type CPT-1 (*Mcpt-1*) is a protein involved in fatty acid metabolism and the expression is regulated by PPARs, such as *Pparα* in cardiomyocytes [31]. We have found that the level of *Mcpt1* mRNA was significantly lower in GK rats than in Wister rats (27±2.6%, p<0.01) (Fig 5). Administration of the mitochondrial nutriments to the GK rats significantly increased the levels of *Mcpt-1* (97±18.4%, p<0.05 vs. GK control) in soleus muscle.

## Discussion

A recent study indicated that mitochondrial dysfunction is present in the prediabetic state [32], suggesting that mitochondrial dysfunction may play a role in the development to 2 type diabetes [3]. The GK rat is a model of non-obese, spontaneous type II diabetes. The pathogenesis of diabetes in the GK rat involves an impaired insulin secretion, insulin resistance, an abnormal glucose metabolism as well as an impaired ontogenetic development of pancreatic islet cells [33], [34] and mitochondrial dysfunction in the liver [35] and heart [36]. In the present study, we observed that defect of glucose and lipid metabolism is associated with low mitochondrial content and reduced mitochondrial enzyme activity in skeletal muscle of the diabetic Goto-Kakizaki rats. We have demonstrated that a combination of 4 mitochondrial nutrients, comparable to the anti-diabetic drug pioglitazone, ameliorated the symptoms of diabetes including beta-cell dysfunction, enhanced mitochondrial biogenesis, improved mitochondrial function and fatty acid and glucose metabolism in diabetic G-K rats. In addition, the pioglitazone caused gain of body weight, a known clinical drawback of thiazolidinedione for treatment of type 2diabetic patients [23]. On contrary, the nutrient treatment did not cause any significant change of body weight, suggesting there is an advantage of nutrients over the anti-diabetic drugs.

The combination contains LA, ALC, biotin and niacin. First, LA has been shown to mitigate insulin resistance in GK rats [37], and it has also been shown that improvement of insulin sensitivity is mediated by activation of AMPK and reduced triglyceride accumulation in skeletal muscle [38].

Second, ALC is the acetyl derivative of L-carnitine which plays an important role in lipid metabolism, acting as an obligatory cofactor for beta-oxidation of fatty acids by facilitating the transport of long-chain fatty acids across the mitochondrial membrane as acylcarnitine esters. Both L-carnitine and ALC are shown to be effective in improving insulin-mediated glucose disposal either in healthy subjects or in type 2 diabetic patients with two possible mechanisms: regulating acetyl and acyl cellular trafficking for correctly meeting energy demand and controlling the synthesis of key glycolytic and gluconeogenic enzymes [39], [40].

Third, biotin-dependent carboxylases play important role in mitochondrial function because four of the five biotin-dependent carboxylases are in the mitochondria. A high intake of biotin may exert effects on beta cells, liver and skeletal muscle, that favor good glucose tolerance [41]. In addition, it was shown that LA could reduce the activities of biotin-dependent carboxylases, such as pyruvate carboxylase and β-methylcrotonyl-CoA carboxylase, in rat liver while biotin co-treatment with LA could normalize these carboxylase activities [42]. Though the mild decreases in carboxylase activities caused by LA would presumably not cause pathology, it is always essential to keep homeostasis and avoid side-effects by a simple co-administration with biotin.

Fourth, niacin has been used in the treatment of cardiovascular diseases for improvement of disturbed lipid and lipoprotein metabolism. It is still the most efficacious drug in terms of its ability to increase HDL cholesterol content accompanied by a decrease in all atherogenic lipoproteins as well as fatty acids and triglycerides. Niacin was shown to be an antilipolytic agent because it, similar to the adenosine receptor agonist phenylisopropyladenosine, lowers plasma glucose, plasma FFA, hepatic glucose production, and enhances insulin-stimulated glucose uptake in streptozotocin-induced diabetic rats [43]. There are studies on niacin against the loss of beta cell function in type 1 diabetes [44], [45]. Nicotinamide injection could prevent beta cell abnormalities, glucose-stimulated insulin secretion loss, GLUT2 loss, and triglyceride accumulation in obese Zucker diabetic fatty rats [46]. Nicotinamide was shown to be effective agent for prevention and treatment of insulin-dependent diabetes mellitus (IDDM) in prediabetic and early stage IDDM because nicotinamide treatments (intramuscularly or orally) normalized antioxidant enzyme system activity and the levels of lipid peroxidation products, and prevented the occurrence of streptozotocin-induced diabetes mellitus in rats [47], [48]. The main reason for us to include this B vitamin is mainly to use it as a source of the NADH and NADPH coenzymes required for mitochondria. NAD (P) H acts as a donor of hydrogen anion in a variety of enzymatic processes, such as the reduction of GSSG to GSH. Increased NAD(P)H may help the reduction of the exogenously administered oxidized form LA , which needs to be NAD(P)H-dependently reduced in mitochondria or cytosol [49]. In addition, Kirsch and De Groot [50] have proposed that NAD(P)H may also act as directly operating antioxidant, which limit the action of freely diffusing radicals by scavenging the attacking, oxidizing radical and reducing oxidized bio-molecules.. Of course, nicotinamide may also improve pancreatic islet dysfunction, which is an important feature of GK pathogenesis [33], [34].

We have recently examined the effect of LA and ALC as well as their combination, on mitochondrial biogenesis in 3T3-L1 adipocyte. We found that treatments with the combination of LA and ALC at concentrations of 0.1, 1, and 10 µM for 24 hrs significantly increased the number of viable mitochondria, expression of mitochondrial DNA, mitochondrial complexes, oxygen consumption, and fatty acid oxidation in 3T3L1 adipocyte. These changes were accompanied with an increase in the mRNA expression of *Pparγ, Pparα,* and *Cpt-1*, and the expression of several transcription factors involved in mitochondrial biogenesis, *Ppargc1α,Tfam,* and *Nrf1* and *Nrf2*. In contrast, the treatments with only LA or ALC alone at the same concentrations showed little effect on mitochondrial function and biogenesis [22]. From these results, we have concluded that combination of mitochondrial targeting nutrients/compounds may complementarily promote mitochondrial synthesis and adipocyte metabolism and possibly prevent and treat insulin resistance in type 2 diabetes.

No study has been carried out on the effect of these nutrients on mitochondrial biogenesis in diabetic animals. Based on our intriguing results of the combination of LA and ALC on mitochondrial biogenesis in adipocytes, the main purpose of the present study is to see whether the in vitro effect of LA and ALC on mitochondrial function and biogenesis could be replicated in vivo in an animal model. As shown in our results, the treatment with the combination of the mitochondrial nutrients stimulated mitochondrial biogenesis through the activation of the *Ppargc1α* pathway, similar to that in 3T3L1 adipocyte. It might be important to perform a study with the individual nutrients on mitochondrial biogenesis in the future; however, from the results we obtained in this in vivo study, we could presume that mitochondrial biogenesis may be mainly induced by the combination of LA and ALC. Biotin may play its physiological role as a prosthetic group in the biotin-dependent carboxylases to compete with LA to keep the homeostasis of mitochondrial carboxylases [42]. Nicotinamide may synergize the effect by providing NAD(P)H for a more reducing environment and enhancing mitochondrial antioxidant defense, thus, improves mitochondrial function leading to better glucose tolerance and maintenance of effective beta cell function.

Although we have termed these nutrients mitochondrial targeting agents and focused on their effects on mitochondria, it should be borne in mind that the four nutrients used are also essential for non-mitochondrial cell functions as well. For example, although lipoic acid is a mitochondrial co-factor and mainly located in mitochondria, it is also available to and influences many activities in other parts of the cell when supplied exogenously. Therefore, there is a possibility that the outcomes are mediated via other cellular organelles and the interactions between mitochondria and other organelles, such as endoplasmic reticulum. Future studies on the multiple functions of these nutrients, such as the effects on endoplasmic reticulum and the interactions with mitochondria are warranted.

We have also tested the effect of much higher doses of these nutrients (10 fold) and found that the higher dose, unlike the lower dose, did not show significant effects in most of the parameters examined. The reason for the loss of the effect of higher doses is unknown but we propose that the nutrients may have a bell-shape curve of dose-dependent effect as most nutrients and drugs. The higher dose may fall on the right side of the bell-shape curve. The loss of the effect might be due to a compromise between the effect and the toxicity. Though further study is needed, the present data of the high dose suggest these nutrients are safe even at 10 fold doses of their effective doses.

In conclusion, a treatment with a combination of 4 mitochondrial nutrients in spontaneous diabetic GK rats, as effectively as the anti-diabetic drug pioglitazone, improved glucose tolerance, insulin release, fat acid metabolism, and mitochondrial function through a stimulation of *Ppargc1α* regulated mitochondrial biogenesis pathway. In addition, the treatment with nutrients is better than with pioglitazone because the nutrient treatment, unlike pioglitazone, did not cause body weight increase. These results suggest that a combination of mitochondria-targeting nutrients may have clinical application for preventing and treating metabolic syndromes, such as diabetes.

## Materials and Methods

### Animals

Four-week-old male diabetic GK rats together with age-matched male non-diabetic Wistar rats were purchased from SLAC Laboratory Animal Co. Ltd (Shanghai, China). All animals were housed at 23±2°C under 12-h light and dark cycles, and allowed access to food and water ad libitum. The experiments were performed in accordance with the Guidelines for Animal Experiments of the Institute for Nutritional Sciences, Chinese Academy of Sciences.

### Material

Anti-tubulin from Sigma (St. Louis, MO, USA); anti- PPARGC1A from Santa Cruz (Heidelberg, Germany); anti-OxPhos Complex I (NADH ubiquinol oxidoreductase 39-kDA subunit 1∶2000), anti-OxPhos Complex II (succinate-ubiquinone oxidoreductase 70-kDA subunit 1∶2000) from Invitrogen (Carlsbad, USA); Reverse Transcription System kit from Promega (Manheim, Germany); HotStarTaq from Takara (Otsu, Shiga, Japan); Nrf1, Pparα, Pparδ, Mcpt-1, D-loop and 18S rRNA primers were synthesized by Bioasia Biotech (Shanghai, China), and ALC (hydrochloride salt) from Sigma Tau (Pomezia, Italy). Biotin and nicotinamide were from Boya Biotech (Shanghai, China), R-α-lipoic acid (tris salt) was a gift from Dr. K. Wessel, Viatris, Germany. Pioglitazone was a gift from Taian Pharmaceutical. Co., Ltd (Shandong, China). TRIzol and other reagents were from Invitrogen (Carlsbad, USA).

### Experimental protocol

Four groups (n = 12 in each group) of rats were used: two control groups: Wistar and GK, and two GK experimental groups with treatments: a pioglitazone group received pioglitazone 10 mg/kg/day by gavage and a nutriment group received a combination of R-LA 50 mg/kg/day, ALC 100 mg/kg/day, biotin 0.1 mg/kg/day and nicotinamide 15 mg/kg/day by gavage. The control groups were received same gavage volume, but saline alone, as administered to the experimental groups. The treatments were started at 6-weeks of age, and continued for 3 months. At approximately 18 weeks of age, animals were anesthetized with an intraperitoneal injection of sodium pentobarbital (60 mg/kg) and sacrificed for obtaining soleus muscle tissue.

### Oral glucose and insulin tolerance test

An oral glucose tolerance test (OGTT, 5 g/kg body weight) was performed after starting nutriment administration following the previous GK rat study [51]. All rats fasted overnight before OGTT. Blood was taken from the retrobulbar vein at 0, 30, 60, 120 and 180 min after the oral glucose administration. Plasma glucose concentrations were determined by the glucose oxidase method.

### Insulin assay

Plasma insulin levels were determined by Rat Insulin-specific RIA kit (Linco Research, Inc., St. Charles, MO) [52].

### Plasma free fatty acid (FFA) measurement

Standard solutions of several saturated and unsaturated fatty acids of varying chain length were prepared in chloroform; concentrations were in the range of 10–100 µmol/l. Four-milliliter portions of the various solutions were placed in 15-ml. stoppered tubes; 2.0 ml of the copper reagent added; and the tubes shaken thoroughly for 2 min. After allowing the layers to separate, the upper, cooper-rich aqueous layer was removed with a fine Pasteur pipette, leaving no traces of the aqueous layer on the surface of the chloroform layer containing the copper soap of the acid. This organic layer must not be contaminated by traces of the copper-containing aqueous solution. Exactly 3.0 ml of the chloroform layer was then transferred to a spectrophotometer cuvette of 1-cm 1ight path, and 0.5 ml of the sodium diethyl dithiocarbamate color reagent added. The contents were mixed by rinsing back and forth from a pipette. The absorbance at 440 nM was measured; a sample of chloroform which had been passed through the whole procedure was used as a reagent blank. The results obtained with this series of fatty acid solutions of known concentration were used to prepare a standard calibration curve, from which the concentration of an unknown can be determined [53], [54].

### Isolation of skeletal muscle mitochondria

The soleus muscle, which is composed predominantly of type I muscle fibers rich in mitochondria, was removed from each leg. A first portion was frozen in liquid N_2_ and used for total RNA and protein extraction. A second portion was used immediately for mitochondrial isolation. Soleus muscles were trimmed off fat and connective tissue, chopped finely with a pair of scissors, and used for mitochondrial isolation according to the method of Birch-Machin et al [55]. Each aliquot was rinsed in ice-cold medium A (120 mmol/l NaCl, 20 mmol/l HEPES, 2 mmol/l MgCl_2_, 1 mmol/l EGTA, and 5 g/l bovine serum albumin; pH 7.4) to remove any residual blood. The disrupted muscle was made up to 20 volumes with respect to the original wet weight of tissue with medium A and homogenized with a hand-held borosilicate glass homogenizer. The homogenate was centrifuged at 600×*g* for 10 min at 4°C. The pellet obtained after centrifugation was resuspended in 8 volumes of medium A and centrifuged (600×*g*, 4°C, and 10 min). The 2 supernatant fluids were combined and were subsequently recentrifuged at 17000×*g* for 10 min at 4°C. The pellet containing the mitochondria was resuspended in 10 volumes of medium A and then centrifuged at 7000×*g* for 10 min at 4°C. The pellet obtained after the last centrifugation was resuspended in 10 volumes of medium B (300 mmol/l sucrose, 2 mmol/l HEPES, 0.1 mmol/l EGTA; pH 7.4) and recentrifuged (3500×*g*, 10 min, 4°C). The resulting pellet, which contained soleus muscle mitochondria, was suspended in a small volume of medium B and was stored at −70°C until analyzed.

### RNA isolation and reverse transcription PCR

Total RNA was isolated from ∼30 mg of tissue using the single-step TRI reagent and 1 ug of RNA was reverse transcribed into cDNA. In brief, the isolated RNA was dissolved in sterile water and 2.5 mmol/l Mg^2+^, 1 mmol/l dNTPs, 0.5 µg oligodT_15_, 25 U AMV reverse transcriptase, 10×RT buffer, giving a final volume of 20 µl. The sample was incubated at 25°C (10 min), 42°C (60 min), and 99°C (5 min). cDNA was diluted in DNase-free water (1∶25) before quantification by real-time PCR. The primers for quantization of mRNA by real-time qPCR for *Nrf1*, *Tfam*, *Ppara*, *Pparδ*, *Mcpt-1*, and 18S rRNA were listed as below:

*Nrf1:* Forward: 5′-TTACAGGGCGGTGAAATGAC-3′,
  Reverse: 5′- GTTAAGGGCCATGGTGACAG-3′;
*Tfam:* Forward: 5′- CCCTGGAAGCTTTCAGATACG-3′,
  Reverse: 5′- AATTGCAGCCATGTGGAGG-3′;
*Ppara:* Forward: 5′- TCACACAATGCAATCCGTTT-3′,
  Reverse: 5′-GGCCTTGACCTTGTTCATGT-3′;
*Pparδ:* Forward: 5′- GCAGATGGGCTGTGATGG-3′,
  Reverse: 5′-ACTGACACTTGTTGCGGTTC -3′;
*Mcpt-1:* Forward: 5′-CATGGTGAACAGCAACTATTACG-3′,
  Reverse: 5′-CATCTGGTAGGAGCACATGG-3′;
18S rRNA: Forward: 5′-CATTCGAACGTCTGCCCTATC-3′,
  Revese: 5′-CCTGCTGCCTTCCTTGGA-3′;

Quantitative PCR was performed in Mx3000P Real-Time PCR system (Stratagene). Each quantitative PCR was performed in triplicate. The rat 18S rRNA gene served as the endogenous reference gene. The evaluation of relative differences of PCR product among the treatment groups was carried out using the ΔΔCT method. The reciprocal of 2CT (used CT as an exponent for the base 2) for each target gene was normalized by that for 18S rRNA, followed by the comparison with the relative value in control cells. Final results were presented as percentage of control.

### Immunoblot analysis of PPARGC1A, complex I and complex II

For protein blots, approximately 100 mg muscle was homogenized in an ice-cold solubilization buffer containing 65 mmol/l Tris (pH 7.4), 150 mmol/l NaCl, 5 mmol/l EDTA, 1% (v/v) Nonidet P-40, 0.5% sodium deoxycholate, 0.1% sodium dodecyl sulfate, 10% glycerol, 1 µg/ml aprotinin, 1 µg/ml leupeptin, 10 mmol/l sodium fluoride, 1 mmol/l Na_3_VO_4_, and 1 mmol/l phenylmethylsulfonylfluoride. Protein concentration was determined using the Bio-Rad DC protein assay. The soluble lysates (10 µg per lane) were subjected to 10% SDS-PAGE; proteins were then transferred to nitrocellulose membranes and blocked with 5% non-fat milk/TBST for 1 h at room temperature. Membranes were incubated with primary antibodies directed against PPARGC1A (1∶1000), tubulin (1∶5000), complex I (1∶2000), and complex II (1∶2000), in 5% milk/TBST at 4°C overnight. After washing membranes with TBST three times, membranes were incubated with horseradish peroxidase-conjugated antibody for 1 h at room temperature. Western blots were developed using ECL (Roche Manheim, Germany) and quantified by scanning densitometry.

### Total DNA isolation and real-time PCR

Total DNA was extracted using the QIAamp. DNA Mini kit, and quantitative (Q) PCR was performed using mitochondrial DNA and genomic NA-specific primers. The rat 18S rRNA gene served as the endogenous reference gene. Melting curves were obtained to ensure specific amplification. The standard curve method was used for relative quantification. Final results are expressed as N-fold differences in mitochondrial D-loop expression relative to the 18S rRNA gene [22].

### Assays for activities of mitochondrial complex I and complex II

Mitochondria were isolated by differential centrifugation of the tissue homogenates. NADH-CoQ oxidoreductase (Complex I) and succinate-CoQ oxidoreductase (complex II) were assayed spectrometrically using the conventional assays with minor modifications [56], [57]. NADH–CoQ oxidoreductase (complex I) activity was assayed by monitoring the reduction of 2,6-dichloroindophenol indophenol (DCPIP) at 600 nm upon addition of assay buffer (10X buffer containing 0.5 mol/l Tris–HCl, pH 8.0, 1% BSA, 10 mmol/l antimycin A, 2 mmol/l NaN_3_, 0.5 mmol/l coenzyme Q1). Final concentration of mitochondria protein was 25 mg/ml. Reaction was started by adding 200 mmol/l NADH and scanned at 600 nm for 2 min. Rotenone (3 mmol/l) was added into the reaction system as blank control. Briefly, complex II was assayed in the assay buffer (10Xbuffer contain 0.5 mol/l phosphate buffer, pH 7.8, 1% BSA, 10 mmol/l antimycin A, 2 mmol/l NaN3, 0.5 mmol/l coenzyme Q1) with mitochondria (final concentration 25 mg/ml). The reaction was started with 10 mM succinate and scanned at 600 nm for 2 min at 30°C.

### Statistics

All values are expressed as means±SEM. Significances of differences among groups and within groups were evaluated using repeated measures ANOVA and the paired Student's *t* test, respectively. A *p* value of less than 0.05 was considered statistically significant.
